# Author Correction: Scenarios of future Indian electricity demand accounting for space cooling and electric vehicle adoption

**DOI:** 10.1038/s41597-021-00996-7

**Published:** 2021-07-30

**Authors:** Marc Barbar, Dharik S. Mallapragada, Meia Alsup, Robert Stoner

**Affiliations:** Massachusetts Institute of Technology, Cambridge, 02139 USA

**Keywords:** Energy supply and demand, Energy efficiency

Correction to: *Scientific Data* 10.1038/s41597-021-00951-6, published online 15 July 2021

The borders of the States and Regions depicted in Figure 1 have been amended at the authors’ request. This has no impact on the quantitative results of the manuscript.

Springer Nature remains neutral with regard to jurisdictional claims in published maps and institutional affiliations.

