# Supplementary material for: Scenarios of future Indian electricity demand accounting for space cooling and electric vehicle adoption
Source: Sci Data. 2021 Jul 15;8:178. doi: 10.1038/s41597-021-00951-6 (PMC8282627; doi:10.1038/s41597-021-00951-6)
Supplement: Supplementary file 1 — Supplementary Information [file 41597_2021_951_MOESM1_ESM.pdf]

List of Figures

1 India's GDP curve-fit and forecasting to 2050 . . . 2
2 Residential survey categorized hourly demand profile . . . 2
3 Commercial survey categorized hourly demand profile . . . 2
4 Normalized sample charging profile schemes . . . 3
5 2050 cooling demand contribution to peak results comparison with IEA's Future of Cooling . . . 3
6 Cooling demand contribution to peak demand . . . 3
7 Results comparison with stated policy World Energy Outlook projections . . . 4
8 Results comparison with sustainable policy World Energy Outlook projections . . . 4
9 Results comparison with Brookings India 2030 projections . . . 4
10 Electric Vehicle demand results comparison with IEA's Global EV Outloook . . . 5

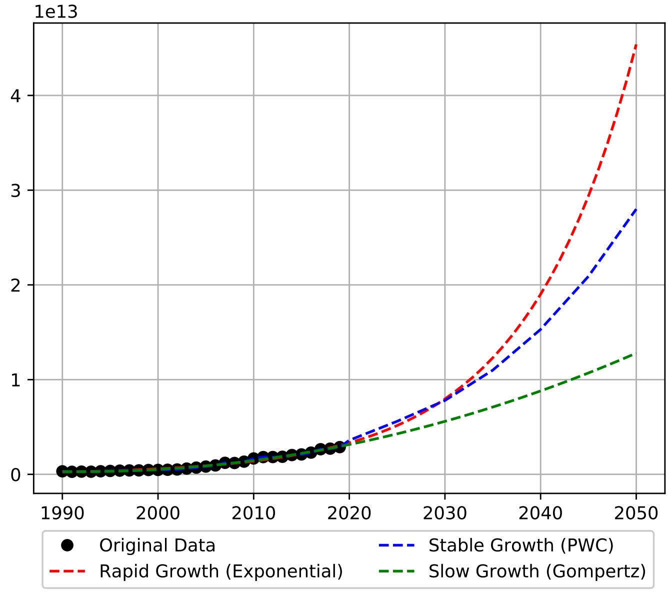

Figure 1. India's GDP curve-fit and forecasting to 2050

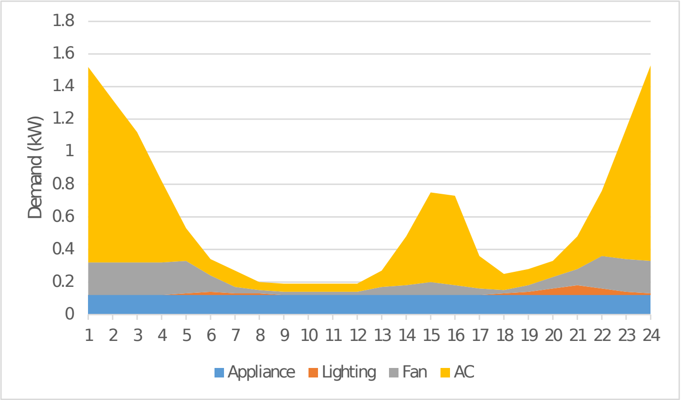

Figure 2. Residential survey categorized hourly demand profile

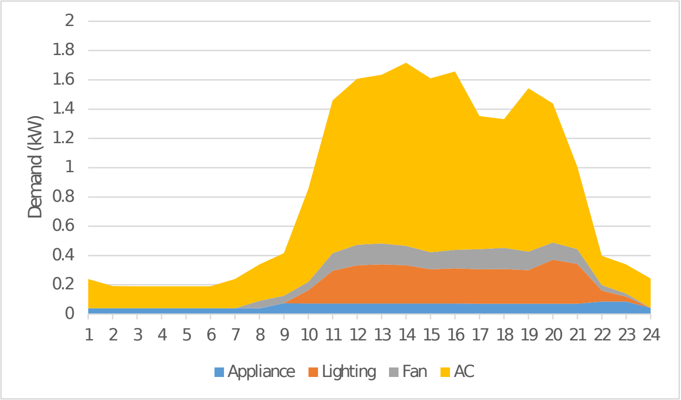

Figure 3. Commercial survey categorized hourly demand profile

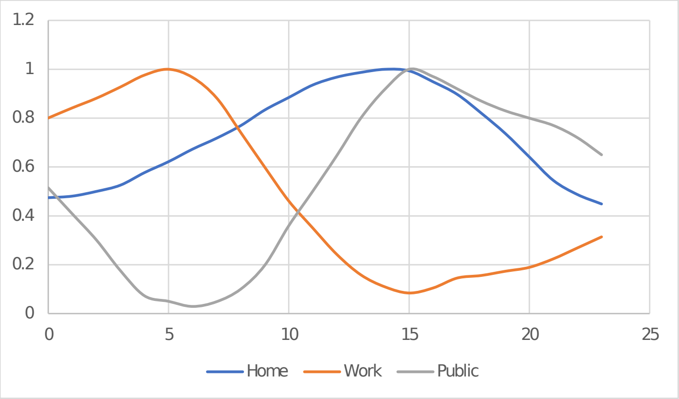

Figure 4. Normalized sample charging profile schemes

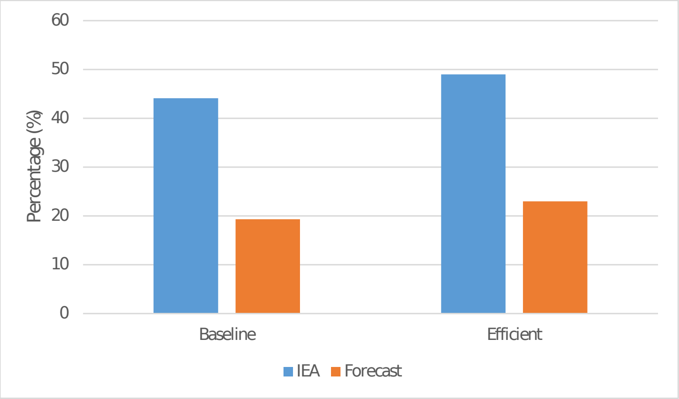

Figure 5. 2050 cooling demand contribution to peak results comparison with IEA's Future of Cooling

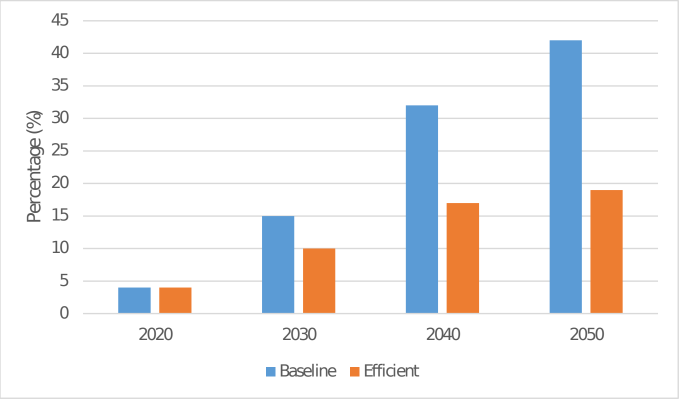

Figure 6. Cooling demand contribution to peak demand

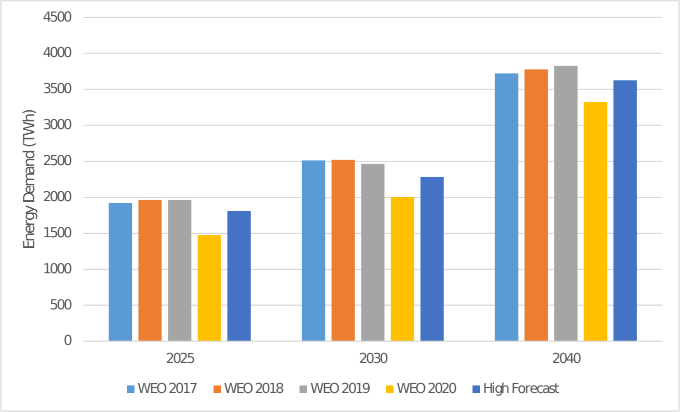

Figure 7. Results comparison with stated policy World Energy Outlook projections

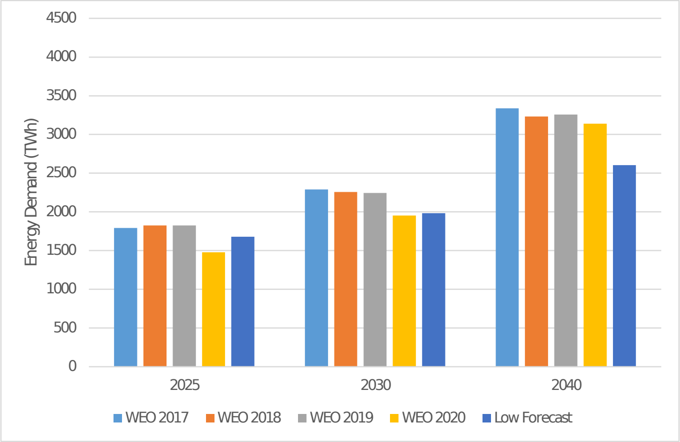

Figure 8. Results comparison with sustainable policy World Energy Outlook projections

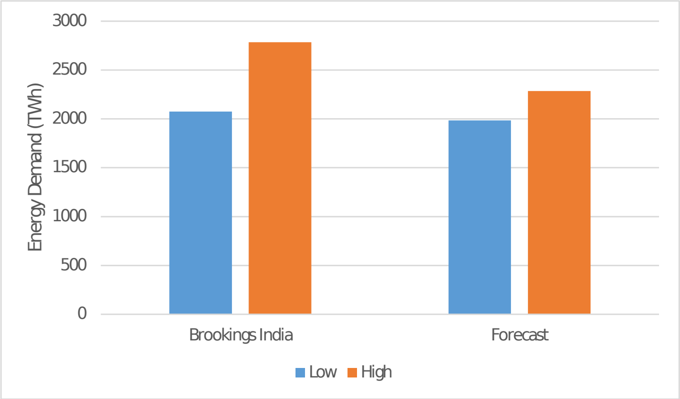

Figure 9. Results comparison with Brookings India 2030 projections

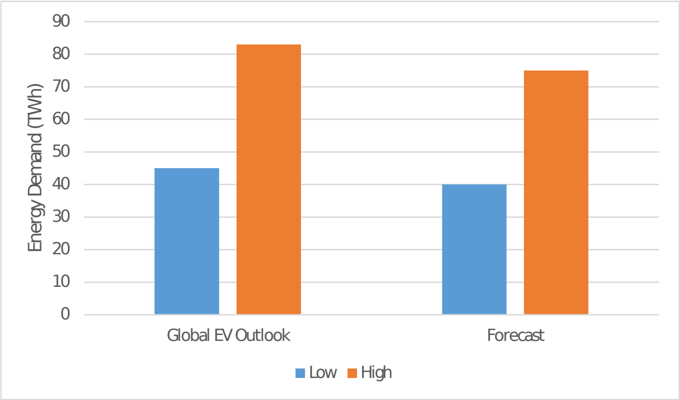

Figure 10. Electric Vehicle demand results comparison with IEA's Global EV Outloook
